# Quantitative neuromagnetic signatures of aberrant cortical excitability in pediatric chronic migraine

**DOI:** 10.1186/s10194-016-0641-x

**Published:** 2016-04-26

**Authors:** Kimberly A. Leiken, Jing Xiang, Emily Curry, Hisako Fujiwara, Douglas F. Rose, Janelle R. Allen, Joanne E. Kacperski, Hope L. O’Brien, Marielle A. Kabbouche, Scott W. Powers, Andrew D. Hershey

**Affiliations:** Division of Neurology, Cincinnati Children’s Hospital Medical Center, 3333 Burnet Avenue, MLC 2015, Cincinnati, OH 45220 USA; Department of Pediatrics, University of Cincinnati, College of Medicine, Cincinnati, OH USA; Division of Behavioral Medicine and Clinical Psychology, Cincinnati Children’s Hospital Medical Center, Cincinnati, OH USA

**Keywords:** Migraine, Magnetoencephalography (MEG), Pediatric, High frequency oscillations, Deep brain sources, Wavelet transform

## Abstract

**Background:**

Reports have suggested that abnormal cortical excitability may be associated with acute migraines. The present study quantitatively assesses the degree of cortical excitability in chronic migraine as compared to acute migraine and healthy controls within the pediatric population.

**Methods:**

We investigated 27 children suffering from chronic migraine, 27 children suffering from acute migraine, and 27 healthy controls using a magnetoencephalography (MEG) system, recording at a sampling rate of 6000 Hz. All groups were age-matched and gender-matched. Neuromagnetic brain activation was elicited by a finger-tapping motor task. The spatiotemporal and spectral signatures of MEG data within a 5–2884 Hz range were analyzed using Morlet wavelet transform and beamformer analyses.

**Results:**

Compared with controls, the chronic migraine group showed (1) significantly prolonged latencies of movement-elicited magnetic fields (MEFs) between 5 and 100 Hz; (2) increased spectral power between 100 and 200 Hz, and between 2200 and 2800 Hz; and (3) a higher likelihood of neuromagnetic activation in the ipsilateral sensorimotor cortices, supplementary motor area, and occipital regions. Compared with acute migraine group, chronic migraine patients showed (1) significantly higher odds of having strong MEFs after 150 ms; and (2) significantly higher odds of having neuromagnetic activation from the deep brain areas.

**Conclusions:**

Results demonstrated that chronic migraine subjects were not only different from the healthy controls, but also different from acute migraine subjects. The chronification of migraines may be associated with elevated cortical excitability, delayed and spread neural response, as well as aberrant activation from deep brain areas.

## Background

The prevalence rate of chronic migraine in the general population is 2–4 % [1]. Each year, approximately 2.5 % of patients with episodic migraines eventually develop new-onset chronic migraines [2]. Migraines are defined as “chronic” when a patient has headaches on 15 or more days per month with migraine features for at least 3 months [3–11]. Diagnoses come, in part, from subjective descriptions of patients’ episodic headache or pain, leaving few clues for objectively investigating the cerebral mechanism underlying chronic migraine.

Recent reports have found that the alteration of cortical excitability plays a key role in *acute* migraine [12–16], crucial information for diagnosis and treatment. However, it remains unclear whether there is a similar association between cortical abnormalities and *chronic* migraines. Therefore, the present study will address this. Magnetoencephalography (MEG) is well suited for objectively investigating the cerebral mechanisms underlying migraine for several reasons [17–19]. First, MEG has outstanding temporal resolution to capture brain signals from low- to very high-frequency ranges [20, 21], superior to the capabilities of functional magnetic resonance imaging (fMRI). Therefore, MEG can be used to reveal the abnormality of brain activity in migraine with millisecond-level resolution. Second, MEG has high spatial resolution to assess where and to what degree altered cortical excitability is occurring in migraine, which is superior to that of electroencephalography (EEG) [22].

The objective of the present study was to quantitatively assess the spatiotemporal and spectral signatures of aberrant brain activation in chronic migraine during headache attacks with MEG. To better understand the spatiotemporal and spectral features of chronic migraine, we compared MEG data from chronic migraine subjects to acute migraine subjects and healthy controls. To our knowledge, this is the first report using both low- and high-frequency neuromagnetic signals to spatially and spectrally assess cortical excitability in pediatric chronic migraine. This study provides quantitative information about the location and nature of the neuromagnetic abnormalities associated with chronic migraine that are not present in typically developing children. Furthermore, this study also reveals neuromagnetic biomarkers for distinguishing chronic migraine from acute migraine. These findings are useful for refining existing treatments, for developing novel diagnosis and treatment protocols for migraines, and for determining preventative strategies for the chronification of migraines [23–27].

## Methods

### Subjects

Twenty-seven chronic migraine subjects (15 females, 12 males; mean (*μ)* = 13.5 years; standard deviation (SD) = ±3.1 years), 27 acute migraine subjects (19 females, 8 males; *μ* = 14.2 years; SD = ±2.4 years), and 27 healthy controls (15 females, 12 males; *μ* = 13.4 years; SD = ±3.2 years) were recruited from the Headache Center at Cincinnati Children’s Hospital Medical Center (CCHMC). Inclusion criteria for migraine participants were: (1) migraine with or without aura as defined in the International Classification of Headache Disorders, 2^nd^ Edition (ICHD-II) [28, 29]; (2) no other neurological disorder and (3) being naïve to medication. Controls were recruited to match the subjects diagnosed with chronic migraine for age and gender. Control subjects met inclusion criteria of: (1) healthy without history of neurological disorder, headache or brain injury; (2) age-appropriate hearing, vision, and hand movement. Exclusion criteria for all participants were: (1) presence of an implant, such as a cochlear implant, a pacemaker or neuro-stimulator, or any other devices containing electrical circuitry, generating magnetic signals, or having other metal that could produce visible magnetic noise in the MEG data; (2) noticeable anxiety regarding participation (e.g., expressing worry about the tests with noticeable physical trembling or sweating); and/or (3) inability or unwillingness to readily communicate with personnel operating the MEG equipment. The research protocol (IRB#: 2010-2438) was reviewed and approved by the Institutional Review Board (IRB) at CCHMC. Informed consent was obtained from each subject prior to testing.

Both chronic and acute migraine subjects were diagnosed and pre-screened by pediatric neurologists of the CCHMC Headache Center. The clinical characteristics of migraine subjects were preliminarily assessed with a questionnaire that is collected as a routine clinical procedure for the Headache Center’s patients, and has also been employed in previously published research studies [30]. The questionnaire assesses headache frequency, duration, severity, and information about prophylactic and treatment medication [29]. If migraine subjects met the criteria of the present study during screening at their clinical appointment, a MEG researcher would then discuss the study with them and obtain signatures on the IRB consent forms. MEG data from the migraine subjects were recorded during their headache attacks. That is, for patients diagnosed with acute migraine, MEG recordings were performed during acute attacks. As chronic migraine patients are consistently suffering from migraine symptoms, MEG recordings were performed in conjunction with these. For all migraine patients, MEG recordings were performed prior to the initiation of either acute or long-term treatment.

### Sound cue task

Participants were instructed to press a button immediately after hearing a 500Hz square wave tone cue. Similar to previous reports [17, 18], subjects used the index finger that was ipsilateral to the tone presented, while keeping other body parts still, and with eyes open and fixed to an arbitrary target during the tests. A trigger from the response button was sent to the MEG system for each button press to align the time course of the behavioral data with the neuromagnetic recordings. The stimuli consisted of 200 trials of tone-button press pairings; 100 tones per ear, which were presented randomly through plastic tubes and earphones. Stimulus presentation and response recording were performed using BrainX software, which is based on DirectX (Microsoft Corporation, Redmond, WA, USA) [17, 18].

### MEG recording

The MEG signals were recorded in a magnetically shielded room (Vacuum-Schmelze, Hanau, Germany) using a whole head CTF 275-Channel MEG system (VSM MedTech Systems Inc., Coquitlam, BC, Canada) in the MEG Center at CCHMC. The sampling rate of the MEG recordings was 6,000 Hz. An acquisition window was set to 3,000 milliseconds (ms) per trial, with a 2,000 ms pre-trigger baseline window. Data were recorded with a noise cancellation of third-order gradients. Subjects were asked to remain still, but if head movement during a recording was beyond 5 mm, that dataset was not included in analysis, and an additional dataset was recorded.

All subjects were familiarized with the MEG facilities, including the magnetically shielded room, MEG system, and the button box, and then provided the opportunity to opt out of participation. Once subjects agreed to participate in the study, it was reconfirmed that they were free from all possible metals. After that, the subjects laid on a specially designed MEG bed while a researcher attached three fiducial coils to the nasion, left, and right pre-auricular points. These three coils were subsequently activated at different frequencies for measuring participants’ head positions relative to the MEG sensors. Just before the MEG recordings, subjects’ heads were positioned in the MEG dewar (or helmet) and their head position was localized.

### Magnetic resonance imaging (MRI) scan

Three-dimensional (3D) MRI was obtained using a 3T Philips Achieva scanner (Philips Healthcare, 3000 Minuteman Road, Andover, MA). Three fiducial marks were placed in identical locations to the positions of the three coils used in the MEG recordings, to allow for an accurate co-registration of the two data sets. Subsequently, all anatomical landmarks were made identifiable in the MRIs.

### Time-frequency decomposition

**Fig. 1:**
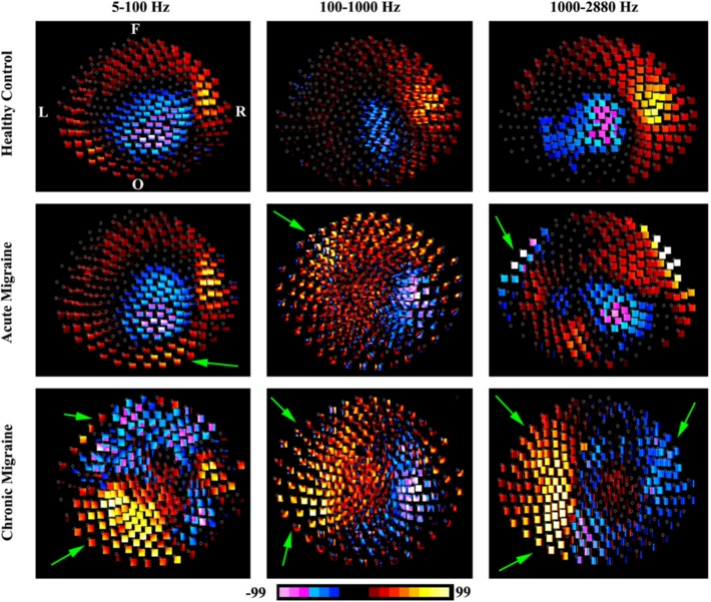
Polarity contour maps of MEG data recorded from a representative chronic migraine subject, acute migraine subject, and control subject during left finger movements. The control subject shows activation predominantly around the primary motor area in all three-frequency bands. The acute migraine subject shows activation around the primary motor cortex as well as other areas (*green arrows*), including the left frontal region and the right occipital area. The chronic migraine subject shows activation out of the primary motor area, which appear to be stronger than the activation from the primary motor areas (*green arrow*). Neuromagnetic in 1000–2884 Hz shows the most disorganized pattern in the chronic migraine subject as compared with the control including activity distributed over the left frontal, left occipital, and right frontal regions. All contour maps are in the same orientation. “L” indicates left; “R” indicates right; “F” indicates frontal; “O” indicates occipital

**Fig. 2:**
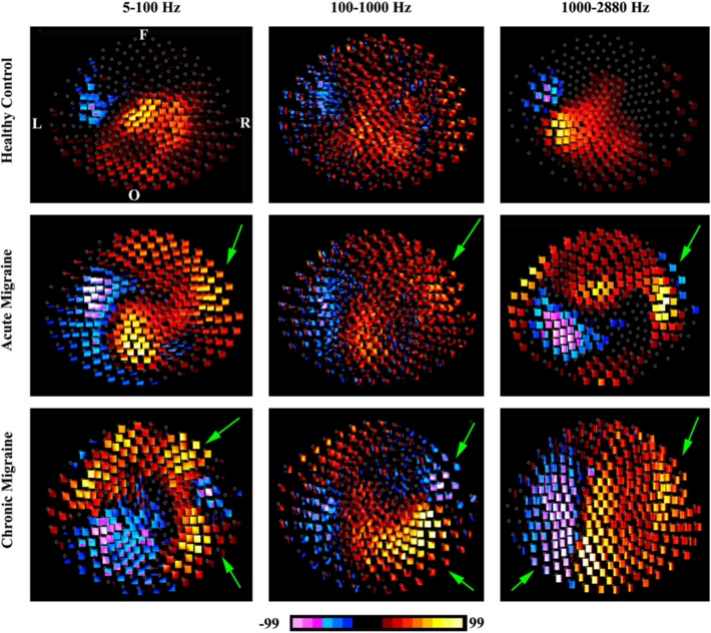
Polarity contour maps of MEG data recorded from a representative chronic migraine subject, an acute migraine subject and a control during right finger movements. The control subject shows activation predominantly around the primary motor area. The acute migraine subject show activation around the primary motor cortex as well as other areas (*green arrows*). The chronic migraine subject shows activation out of the primary motor area, which appear to be overshadow the activation around the primary motor areas (*green arrow*). Neuromagnetic in 1000–2884 Hz shows the most disorganized pattern in the chronic migraine subject as compared with the control. All contour maps are in the same orientation. “L” indicates left; “R” indicates right; “F” indicates frontal; “O” indicates occipital

**Fig. 3:**
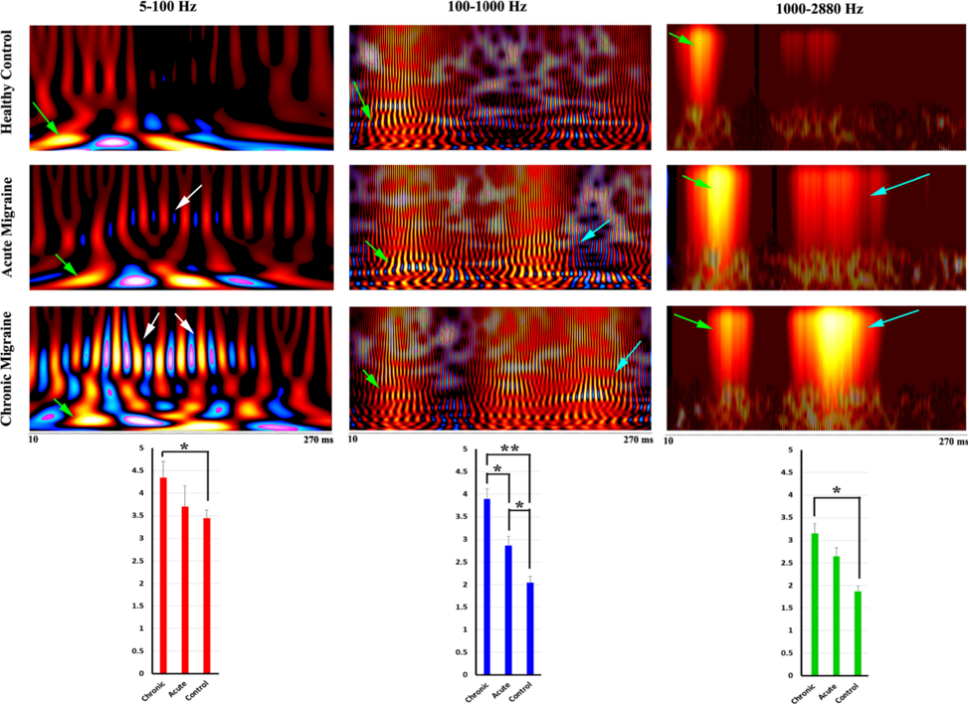
Global spectrograms of MEG data recorded from a representative chronic migraine subject, an acute migraine subject, and a control during left finger movements. The spectrograms in 5–100 Hz show delayed spectral components in acute and chronic migraines (*light green arrows* indicate component 1, *dark green arrows* indicate component 2) as compared with controls. Spectral components around 70–80 Hz (*white arrows*) appear to be strong in chronic migraine, to be weak in acute migraine, and are not identifiable in the control. The spectrograms in 100–1000 Hz and 1000–2880 Hz reveal later components (*cyan arrows*) in chronic and acute migraine, but not in the controls. The vertical axis contains the frequency range (i.e., the minimum frequency at the bottom; maximum on the top). The horizontal axis contains the time window (from 10 ms to 270 ms). Below the spectrograms are bar graphs showing the spectral power of neuromagnetic activation elicited by left finger movements in chronic migraine subjects, acute migraine subjects and healthy controls. The bars show the mean and standard error of the spectral power of each group of subjects. “**” indicates *p* < 0.001; “*” indicates *p* < 0.01

**Fig. 4:**
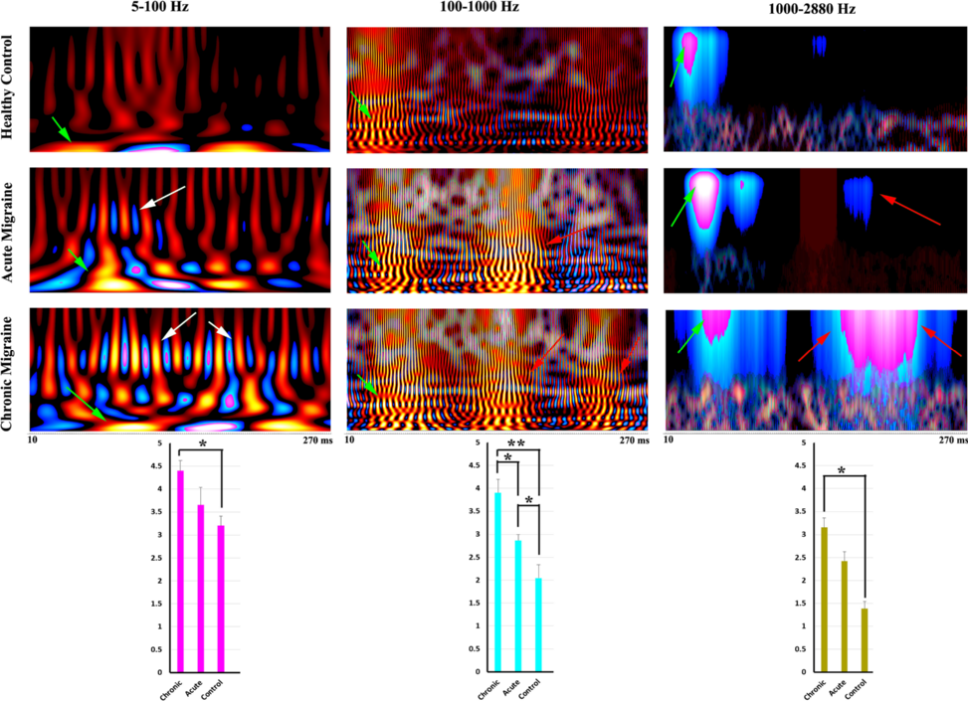
Global spectrograms of MEG data recorded from a representative chronic migraine subject, an acute migraine subject and a control during left finger movements. The spectrograms in 5–100 Hz show delayed spectral components in acute and chronic migraines (*light green arrows* indicate component 1, *dark green arrows* indicate component 2) as compared with controls. Spectral components around 70–80 Hz (*white arrows*) appear to be strong in chronic migraine, to be weak in acute migraine, and are not identifiable in the control. The spectrograms in 100–1000 Hz and 1000–2880 Hz reveal later components (*cyan arrows*) in chronic and acute migraine, but not in the controls. The vertical axis contains the frequency range (i.e., the minimum frequency at the bottom; maximum on the top). The horizontal axis contains the time window (from 10 ms to 270 ms). Below the spectrograms are bar graphs showing the spectral power of neuromagnetic activation elicited by right finger movements in chronic migraine subjects, acute migraine subjects and healthy controls. The bars show the mean and standard error of the spectral power of each group of subjects. “**” indicates *p* < 0.001; “*” indicates *p* < 0.01

MEG waveforms were transformed into spectrograms, the time-frequency representations of MEG data [17, 31], using MEG Processor, a customer-designed program for MEG data analyses [32, 33]. The spectral characteristics of MEG data were analyzed with spectrograms computed with the Morlet continuous wavelet algorithm [32–34]. Since frequency-temporal resolution changes with the “sigma value” (the number of wave cycles completed within a time window), the time-frequency analysis method was improved for this study by dynamically changing the sigma value according to frequency ranges [17, 31]. We analyzed neuromagnetic signals in three frequency ranges, which included 5–100 Hz, 100–1000 Hz and 1000–2884 Hz. We did not include neuromagnetic signals below 5 Hz, as the computation of such low-frequency components requires an extremely high number of data points. Moreover, activity below 5 Hz may include *pre-motor* activation, such as the readiness magnetic fields, which we took care to exclude from our analyses of the motor responses of patients with migraine. For each frequency range, we used 600 frequency bins. Consequently, the frequency resolution was 6.315 ((100–5 Hz)/600); 0.667 ((1000–100 Hz)/600); and 0.318 ((2884–1000 Hz)/600) data points per Hz for the three frequency ranges, respectively. To measure magnetic spectral power elicited by finger movements, accumulated spectrograms from 100 trials for the left finger and 100 trials for the right finger were computed separately. The magnetic polarity of the activity was part of the color-coding scheme of the “polarity spectrogram” (Figs. 1, 2, 3 and 4).

### Sensor level analysis

The polarity spectrograms, which depict the positive and negative directions of the magnetic fields for each MEG sensor, were overlaid onto contour maps of the entire brain for visualizing the spatial distribution of the movement-elicited brain activation [13, 32]. These contour maps were used to demonstrate the source patterns. A global spectrogram (a summation of all sensors) for each condition (for left or for right finger movement) was computed for assessing the spectral components of the entire cortex. To quantify total brain activation that was elicited by movement, the absolute spectral power across sensors was computed using the root-mean-square (RMS) value excluding polarity information. To facilitate the measurements, we developed a toolbox, which could automatically measure the mean value for each frequency bin of all MEG sensors. The automatic measurements of the mean values of spectrograms from all MEG sensors showed less inter-individual variation among controls. The time window for quantifying spectral power at sensor levels was 10–270 ms for all the three frequency ranges. This time window was selected to capture typical movement-related magnetic fields without including the prior readiness fields [13, 14].

### Source level analysis

Wavelet-based beamformer was used in the source analysis [32–34]. That is, in order to capture the dynamic spatiotemporal activity in the brain, we applied a sliding window in the source estimation process. Multiple local spheres were used for magnetic forward computing of the sources of activity. MEG Processor was used to compute and visualize magnetic sources [31, 35]. As described above in the sensor-level analysis, the time window for source estimation was also 10–270 ms for signals in the frequency ranges of 5–100 Hz, 100–1000 Hz and 1000–2,884 Hz. The whole brain was scanned with a 3-millimeter resolution grid. In comparison with our previous reports [31, 35], one methodological improvement in the present study was that the source power and goodness-of-fit (GOF) were computed for each grid position. The details of the methodology has been described in previous reports [32, 34].

### Statistical analysis

The effects of chronic migraine on latency and spectral power were analyzed with Analyses of Variance (ANOVA). The fixed factor was the groups of migraine subjects versus controls. The dependent variables were latency and spectral power. The comparisons of chronic vs. control group, chronic vs. acute group, and acute vs. control group for left and right finger movements were performed with *Student* T-tests. The odds ratios of activity in brain areas other than the primary motor cortex among the chronic, acute, and control groups were analyzed with Fisher’s exact tests. Since subjects performed left and right finger movements, the aforementioned statistical analyses were performed twice, one for each finger. *p* < 0.05 was considered significant for each paired comparison. Bonferroni correction was applied for multiple comparisons (i.e. since three frequency ranges were analyzed, 0.05/3 = 0.016, only *p* < 0.01 was very conservatively considered to be significant).

## Results

### Clinical characteristics

The clinical characteristics are shown in Table 1. Control group participants were recruited to match the chronic migraine group in age, gender, and handedness. There was no headache in the control group. In the chronic migraine group, 15 of the 27 subjects were female, which was identical to the control group demographic, but different from the acute migraine group. The chronic migraine group has significantly higher frequency of headaches per month as compared with the acute migraine group (*p* < 0.01). T-tests show no significant differences between chronic and acute migraine groups in terms of the other parameters, including years of suffering from migraine, duration of headache, and severity of headache (*p* > 0.05). This suggests that differences in neural responses between groups may be associated with headache frequency as opposed to another feature distinguishing groups.

**Table 1:** Demographic and Clinical Characteristics of Subjects

Parameters		Measurements		
		Chronic	Acute	Control
Gender (female/male)		15/12	19/8	15/12
Age (years) (mean ± SD)^a^		13.5 ± 3.1	14.2 ± 2.4	13.4 ± 3.2
Handedness (right/left)		24/3	24/3	24/3
Frequency of headache per month (mean ± SD)		20.3 ± 7.3	7.5 ± 3.6	0
Years of suffering from migraine (mean ± SD)		5.9 ± 4.1	3.8 ± 2.7	0
Duration of headache (hours) (mean ± SD)		9.1 ± 12.3	9.0 ± 4.6	0
Severity of headache (on scale: 0 ~ 10) (mean ± SD)		6.0 ± 1.9	6.5 ± 2.6	0
Pain type (Number of subjects; Multiple descriptions were allowed)	Throbbing	21	17	0
	Pressure	16	13	0
	Constant	12	6	0
	Sharp	6	6	0
	Squeezing	9	3	0
	Stabbing	6	2	0
	Others	1	2	0

^a^ *SD* standard deviation

### Behavioral data

The mean and standard deviation of the button-press response time after hearing the sound cue are shown in Table 2. There was a significant difference between the chronic migraine and control groups in terms of the response time for left finger movement (*p* < 0.05), but not for right finger movement. There was no statistical difference between the chronic migraine and acute migraine groups in terms of the response time for either left or right finger movement.

**Table 2:** Movement Response Time (milliseconds) after Hearing the Sound Cue

	Chronic (Mean ± SD) ^b^	Acute (Mean ± SD)	Control (Mean ± SD)
Left-hand	425.3 ± 183.4^a^	413.8 ± 193.4	364.5 ± 142.6
Right hand	392.4 ± 157.9	387.4 ± 161.6	358.4 ± 146.2

^a^ *P* < 0.05 (Chronic vs. control)

^b^ *SD* standard deviation

### Sensor-level contour map

#### Low-frequency activation (5–100 Hz)

As shown in Figs. 1 (left hand) and 2 (right hand), as expected, the most consistent neuromagnetic activation in 5–100 Hz was identified around the primary motor region in the hemisphere contralateral to the finger pressing the button in the control, chronic and acute migraine groups (i.e. left hemisphere activation in response to right-hand button press, and right hemisphere activation in response to left-hand button press). However, the patient groups, with chronic and acute migraine, also show dispersed neuromagnetic activation outside of the typical primary motor activation (Green arrows in Figs. 1–2). Not only is this additional activity found in migraine groups and not controls, but the intensity of this activity appears to be stronger for chronic migraine subjects as compared to acute migraine subjects.

#### High-frequency activation (100–1000 Hz)

Contour maps revealed that the most consistent neuromagnetic activation in 100–1000 Hz was identified in the primary motor area in the contralateral hemisphere in all three groups of subjects (Figs. 1–2). We noted both chronic and acute migraine subjects showed dispersed neuromagnetic activation around the primary motor region as compared with controls (Green arrows in Figs. 1–2).

#### Very high-frequency activation (1000–2884 Hz)

As in the lower frequency bands, contour maps revealed that the most consistent neuromagnetic activation in 1000–2884 Hz was identified in the primary motor area in the hemisphere contralateral to the finger pressing the button in the control groups (Figs. 1–2). The contour maps in the chronic migraine group were different from that of control and acute migraine groups (Green arrows in Figs. 1–2). The dipolar pattern around the primary motor region, which was consistently presented in control group, was not clearly identifiable in the chronic migraine group (Figs. 1–2).

### Global spectrograms

#### Low-frequency activation (5–100 Hz)

As shown in Fig. 3 (responses to left-hand button press) and Fig. 4 (responses to right-hand button press), global spectrograms in 5–100 Hz revealed movement-elicited oscillatory activation (green arrows) in all three groups. We noted abnormal focal oscillatory increases (cyan arrows in Figs. 3–4) for acute and for chronic migraine subjects, that do not appear for controls. Bar graphs in Figs. 3 and 4 under each frequency band show quantitative measurements of the spectral power of neuromagnetic activation across the whole time window represented in the spectrogram. For both the right and left hand, in this frequency range, chronic migraine patients show significantly higher spectral power than controls (Figs. 3–4).

**Fig. 5:**
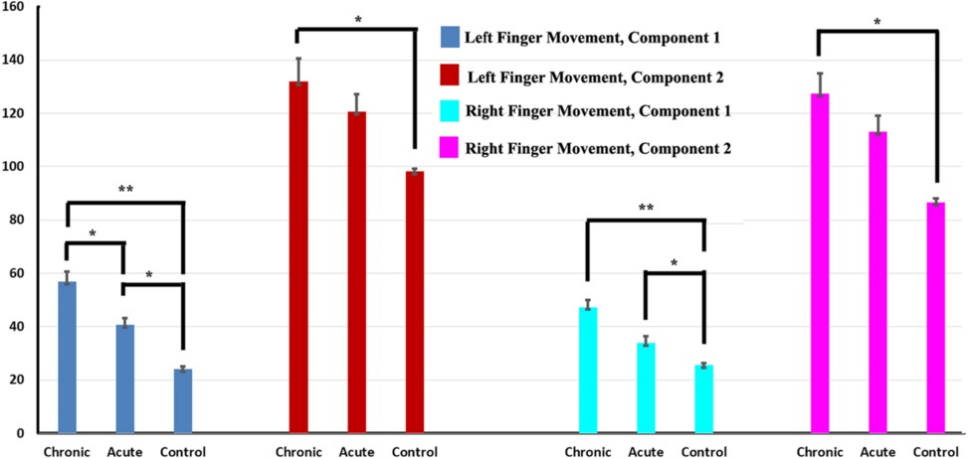
The latencies of the first and second spectral components in 5–100 Hz in chronic migraine subjects, acute migraine subjects, and healthy control subjects. The x-axis measures time in milliseconds. The bars show the mean and standard error of latencies of spectral components in each group of subjects. The double asterisk indicates *p* < 0.001; and a single asterisk indicates *p* < 0.01

While all three groups show 2 early neuromagnetic increases (as indicated by green arrows in the first column of Figs. 3–4), acute and chronic migraines show delays in these responses. Figure 5 shows the exact latencies for each of these early increases in this frequency band. The first response increase, “Component 1”, and the second response increase “Component 2” in the chronic migraine group are significantly slower than controls for both right and left hands, whereas acute migraines only show significant delays compared to controls for both hands in Component 1. Chronic migraines only show significantly delayed responses compared with acute migraines for Component 1 in the left hand. A 3-way ANOVA was run on the total sample of 81 participants to examine the effect of gender, age, and headache frequency on spectral power, and no significant interaction was found in this conventional frequency range.

#### High-frequency activation (100–1000 Hz)

Global spectrograms in 100–1000 Hz revealed movement-elicited high-frequency oscillatory activation (green arrows in Figs. 3–4) in all three groups. Abnormal focal oscillatory increases were also revealed for migraine groups but not for controls (Cyan arrows in Figs. 3–4). Due to the rapid oscillatory activity in this frequency range, latency differences could not be measured. Therefore, in these frequency ranges, differences between groups were detected in terms of spectral power only. The bar graph for the 100–1000 Hz range is shown below the spectrograms for each group in Figs. 3–4. As in the low frequency range, chronic migraines show significantly higher spectral power than controls for both hands. However, in addition to this finding, bar graphs indicate that acute migraines also show significantly higher spectral power than controls, as well as significantly lower spectral power than chronic migraines, for both hands. This suggests that the abnormal motor responses underlying acute migraines are only detectable in higher frequency ranges, whereas as chronic migraine abnormalities are more distributed across the frequency bands. A 3-way ANOVA was run on the total sample of 81 participants to examine the effect of age, gender, and headache frequency on spectral power. A significant effect of migraine frequency (and not age or gender) on spectral power was revealed (*F* (1, 26) = 13.21, *p* = 0.003). This finding suggests that higher frequencies such as these are crucial for detecting oscillatory differences underlying neurological disorders.

#### Very high frequency activation (1000–2884 Hz)

Global spectrograms in the 1000–2884 Hz range revealed movement-elicited very high-frequency oscillatory activation (green arrows in Figs. 3–4) in all three groups. Due to the extreme speed of fluctuations, the individual very high frequency oscillations merged as clusters (See arrows in Figs. 3–4). We therefore measured the spectral power, but not latency. Compared to healthy controls, acute migraine subjects only showed slightly increased late activation. However, chronic migraine subjects showed much stronger late activation as compared to healthy controls and acute migraine subjects (cyan Arrows in Figs. 3–4). A 3-way ANOVA was run on the total sample of 81 participants to examine the effect of gender, age, and headache frequency on spectral power, and no significant interaction was found in this frequency range.

### Magnetic Source Imaging

#### Low-frequency activation (5–100 Hz)

**Fig. 6:**
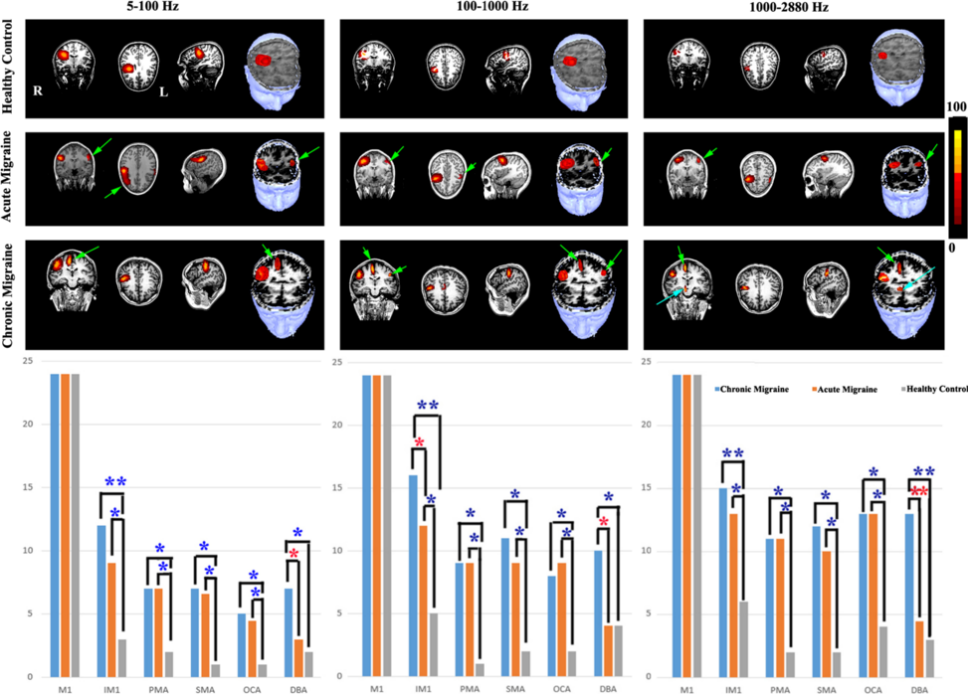
Magnetic source imaging (MSI) showing the locations of neuromagnetic activation in response to left-finger movement in a representative chronic migraine subject, acute migraine subject, and control subject. The primary motor cortex in the hemisphere contralateral to the finger pressing the button is activated in all the three subjects (*no arrows*). The ipsilateral sensorimotor area, supplementary motor area, and the pre-motor areas are activated only in chronic and acute migraine subjects (*green arrows*). The deep brain area is activated only in the chronic migraine subject (*cyan arrows*). In all coronal and axial views, “R” indicates right and “L” indicates left. Below the MSI images are bar graphs indicating the sources of neuromagnetic activation elicited by left finger movements in three groups of subjects. The bars show the number of subjects having activation in each location in each group. M1 indicates primary motor area; IMA indicates ipsilateral motor area; PMA indicates pre-motor area; SMA indicates supplementary motor area; OCA incites occipital area and DBA indicates deep brain area. The odds ratio was statistically analyzed by comparing each group. The double asterisk indicates *p* < 0.001; and a single asterisk indicates *p* < 0.01. A red asterisk indicates a difference between acute and chronic migraines and blue asterisks represent the other group differences

**Fig. 7:**
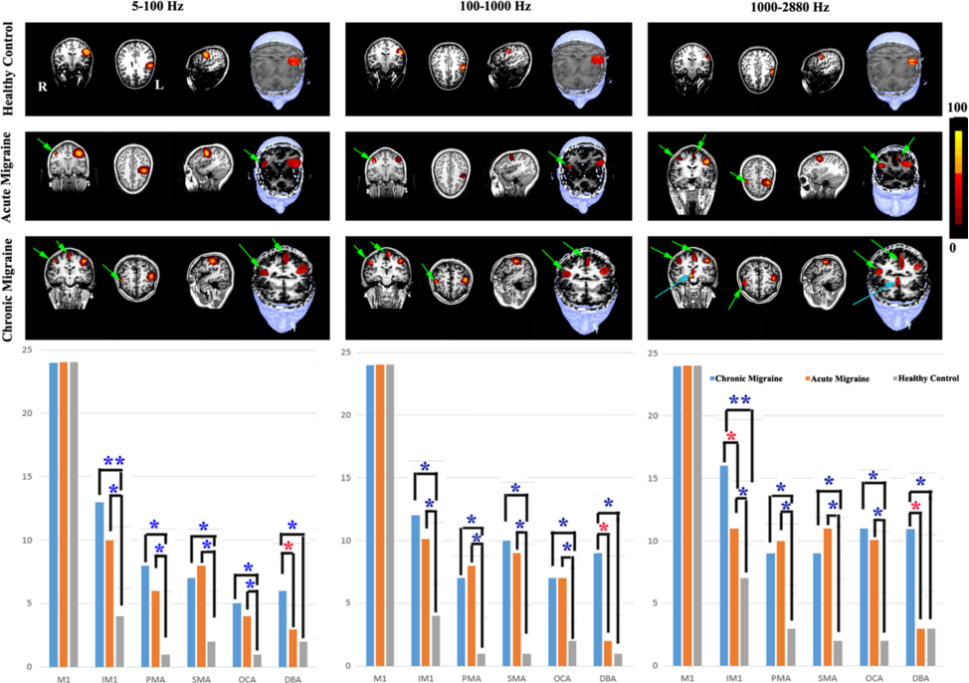
Magnetic source imaging (MSI) showing the locations of neuromagnetic activation in response to right-finger movement in a representative chronic migraine subject, acute migraine subject, and control subject. The primary motor cortex in the hemisphere contralateral to the finger pressing the button is activated in all the three subjects (*no arrows*). The ipsilateral sensorimotor area and the supplementary motor area are activated in chronic and acute migraine subjects (*green arrows*). The deep brain area is activated only in the chronic migraine subject (*cyan arrows*). In all coronal and axial views, “R” indicates right and “L” indicates left. Below the MSI images are bar graphs indicating the sources of neuromagnetic activation elicited by right finger movements in three groups of subjects. The bars show the number of subjects having activation in each location in each group. M1 indicates primary motor area; IMA indicates ipsilateral motor area; PMA indicates pre-motor area; SMA indicates supplementary motor area; OCA incites occipital area and DBA indicates deep brain area. The odds ratio was statistically analyzed by comparing each group. The double asterisk indicates *p* < 0.001; and a single asterisk indicates *p* < 0.01. A red asterisk indicates a difference between acute and chronic migraines and blue asterisks represent the other group differences

The most consistent location of neuromagnetic activation was the primary motor area (M1) in all three groups of subjects (Figs. 6–7). We also noted activation in the ipsilateral motor area (IMA), the premotor area (PMA), the supplementary motor area (SMA), the occipital area (OCA) and the deep brain area (DBA). The odds ratio of the activation in the aforementioned areas are quantified and summarized in bar graphs below each frequency band. For 5–100 Hz, chronic migraines showed significantly higher activation than controls in all of the above listed regions, for both hands. Acute migraines showed significantly higher activation than controls in all regions except for the deep brain area, for both hands. Interestingly, this is also the only region that showed significantly higher activation for chronic migraines as compared with acute migraines, for both hands (as indicated by a red asterisk as opposed to blue). This suggests that the deep brain area in this frequency band may be a biomarker distinguishing the chronification of migraines from acute migraines.

#### High-frequency activation (100–1000 Hz)

The most consistent location of neuromagnetic activation was the M1 in all three groups of subjects (Figs. 6–7). We also noted activation in the IMA, the PMA, the SMA, the OCA, and the DBA. Bar graphs below the MSI images show that in this frequency, chronic migraines have significantly higher activation in each of these regions than controls, for both hands. As in the lower frequencies, this range shows significant activation increases for acute migraines compared with controls in all of the above listed regions except for DBA, in both hands. Of note, in this frequency band this DBA region also shows significant increases for chronic over acute migraine activation, in both hands. Furthermore, the left hand shows significant increases for chronic versus acute in the IMA region.

#### Very High-frequency activation (1000–2884 Hz)

The most consistent location of neuromagnetic activation was the M1 in all three groups of subjects (Figs. 6–7). We also noted activation in the IMA, the PMA, the SMA, the OCA, and the DBA. Bar graphs beneath the MSI figure show significant increases in all of these regions for chronic migraines compared with controls, for both hands. Just as in the lower frequency bands, acute migraines show increases in all areas compared with controls, except in the DBA, where chronic migraines show a significant increase in activation compared with acute migraines, for both hands. There is also a significant increase for chronic versus acute migraines in the IMA for the right hand.

### Frequency signature

A comparison of the contour maps and global spectrograms in three frequency ranges (5–100 Hz, 100–1000 Hz, and 1000–2884 Hz) revealed that there were frequency-dependent neuromagnetic abnormalities in chronic and acute migraine (Figs. 1–4). Very high-frequency signals, 1000–2884 Hz, revealed the most significant abnormalities underlying chronic migraine in terms of in the contour map patterns and the aberrant regions of activation revealed by magnetic source imaging. The 100–1000 Hz range also revealed the most significant spectral power abnormalities of chronic migraine in the global spectrograms. These findings suggest that investigations in frequency bands above 100 Hz are crucial for identifying the brain dysfunction underlying childhood neurological disorders, such as migraine, which is necessary for improved diagnosis and treatment.

## Discussion

MEG data from the three groups of pediatric migraine subjects have demonstrated that there were spatial, temporal, and spectral neuromagnetic differences between pediatric migraine groups versus pediatric control subjects (μ = 13.4 years), as well as between chronic migraine (μ = 13.5 years) and acute migraine (μ = 14.2 years) subjects during a button-press paradigm. We noted that clinical assessment of pediatric acute migraine has been well characterized in previous reports [13, 14, 36, 37]. However, assessment of pediatric chronic migraine seems relatively scarce. While typical diagnoses of chronic migraine require at least 15 headaches per month, the present study found that the average frequency tended to be even higher in the pediatric population investigated here (approximately 20/month).

The analyses of neuromagnetic signals in 5–100 Hz show that in the chronic migraine group the latency of early responses, labeled “Component 1” and “Component 2” here, was significantly delayed as compared with the control group during left and right finger movements. This latency finding is consistent with previous reports using conventional measurements of waveform responses [18]. In addition to confirming prior waveform findings, the spectrograms of the present study provide novel frequency descriptions of neuromagnetic components.

The latency of the first spectral component in the chronic migraine group was also significantly delayed as compared with the acute migraine group, but only in left-finger movements. This phenomenon has not been revealed in any previous report. We expect that this finding results from the fact that the subjects in the migraine groups were predominately right-handed. Consequently, motor cortical dysfunction in chronic migraine during right finger movement may be more easily compensated for in right-handers, but as the left-hand is already weaker in these subjects, the addition of cortical dysfunction perhaps becomes more apparent during left-handed tasks; in this case in terms of latency.

In addition to timing differences, the present study also revealed significant differences between groups in terms of spectral power. Specifically, the neuromagnetic spectral power in 5–100 Hz in chronic migraine subjects was significantly increased compared to controls during left and right finger movements. Similar to previous reports on acute migraine [13, 14], a significant increase of brain activation suggests that the cortical excitability is elevated in migraine. This finding is consistent with previous MEG studies [16, 18] and fMRI studies showing that migraine subjects have greater activation in the primary motor cortex compared to controls [38].

The data of the present study have also revealed that the spectral power of brain activation in 100–1,000 Hz in chronic migraine was significantly higher than that of the acute migraine. Though the exact cerebral mechanism remains unknown, the increase of high-frequency oscillations in 100–1,000 Hz may reflect the activation of the cortical-subcortical networks during the onset of discrete movements, or may signal the direct modulation of outputs from the subthalamic nucleus to the basal ganglia, thereby facilitating movement execution [39]. In fact, the present study and previous publications [13, 14, 40] support the notion of increased cortical excitability in the brain with chronic and acute migraine during headache attacks. Further, these findings suggest that the abnormality in chronic migraine is more severe than that of the acute migraine.

Source analyses revealed that both chronic and acute migraine subjects had distributed brain activation outside of the primary motor area. In comparison to controls, chronic migraine subjects had a significantly higher likelihood of activation in the ipsilateral sensorimotor area, the supplementary motor area, the pre-motor area, the occipital region, and the deep brain area. This observation is similar to many previous reports [41, 42]. The distinct spatial patterns suggest that the dysfunction associated with migraine recruits an abnormally large neural network for a basic motor task.

Analyses of magnetic sources also revealed that chronic migraine had significantly higher odds of having activation in the deep brain area in comparison with acute migraine and controls. Though the cerebral mechanisms underlying aberrant chronic activation remain unclear, we postulate that involvement of the deep brain area may indicate that brain dysfunction in chronic migraine goes beyond the cerebral cortex. From a clinical point of view, aberrant neuromagnetic signals from the deep brain areas may be unique to chronic migraine patients.

MEG signals in the 5–100 Hz, 100–1000 Hz, and 1000–2884 Hz appear to reveal different aspects of migraine-related abnormalities in both chronic and acute migraine, such as response latency, spectral power, and brain regions, respectively. Though previous reports have shown that the brain generates signals around 2632 Hz in the somatosensory cortex [43] and 2500 Hz in the epileptogenic regions [44], those previous reports are based on invasive intracranial recordings, and are limited to patients with intractable epilepsy who are surgical candidates [43, 44]. Therefore, this is the first report showing neuromagnetic signals in this frequency range (>100 Hz) in migraine subjects using *noninvasive* MEG. The results of the present study showed that, neuromagnetic signals can not only differentiate chronic migraine subjects from healthy controls, but also can also reveal differences between chronic migraine patients and acute migraine patients. The difference between chronic migraine and acute migraine may enable us to investigate the chronification of migraine attacks and develop new strategies to prevent acute migraine from developing into chronic migraine.

## Conclusions

In summary, this study has demonstrated that chronic migraine subjects show aberrant neural activation in both low- and high-frequency ranges as compared with controls. The primary novel findings of the present study were (1) the identification of very high-frequency brain activation in chronic migraine subjects using MEG; (2) the determination of increased ictal cortical activation beyond the primary motor cortex in chronic migraine subjects during a button-press task; and (3) the observation of spatiotemporal and spectral differences between chronic and acute migraine. These findings support the notion that chronic migraine is a neurological disorder caused by cortical dysfunction and the evolution from acute migraine is manifested in further increases and delays in cortical and deep brain activation [45, 46].
